# Comparative Evaluation of Oral *Candida albicans* Carriage in Children with and without Dental Caries: A Microbiological *in vivo* Study

**DOI:** 10.5005/jp-journals-10005-1146

**Published:** 2012-08-08

**Authors:** Binita Srivastava, Hind Pal Bhatia, Visuja Chaudhary, Archana Aggarwal, Ashish Kumar Singh, Nidhi Gupta

**Affiliations:** Professor and Head, Department of Pedodontics and Preventive Dentistry Santosh Dental College and Hospital, Ghaziabad, Uttar Pradesh, India; Professor, Department of Pedodontics and Preventive Dentistry Santosh Dental College and Hospital, Ghaziabad, Uttar Pradesh India; Postgraduate Student, Department of Pedodontics and Preventive Dentistry, Santosh Dental College and Hospital, Ghaziabad, Uttar Pradesh, India, e-mail: visujachaudhary@gmail.com; Additional Professor, Department of Pedodontics and Preventive Dentistry, Santosh Dental College and Hospital, Ghaziabad, Uttar Pradesh, India; Reader, Department of Orthodontics and Dentofacial Orthopedics Sardar Patel Post Graduate Institute of Dental Sciences, Lucknow Uttar Pradesh, India; Senior Lecturer, Department of Pedodontics and Preventive Dentistry Santosh Dental College and Hospital, Ghaziabad, Uttar Pradesh, India

**Keywords:** *Candida albicans*, Oral cavity, Caries, Prevalence

## Abstract

**Aim:** The aim of this study was to examine the presence of *Candida albicans* in extensive carious lesions before and after treatment of the carious lesions and to evaluate the carriage of *Candida albicans* in children with and without caries.

**Materials and methods:** The study was conducted on 60 childrens who were divided into two groups: Experimental group (group 1) and controlled group (group 2). Each group was further divided into 3 subgroups according to the dentition as: Group A (Deciduous), group B (Mixed) and group C (Permanent). Swab samples for mycological studies were collected from the dorsum of the tongue, vestibular sulcus and peak of the palatal vault. All samples were cultured directly on SDA plate (Sabouraud's dextrose agar). Number of *Candida* colonies was determined by counting colony forming unit on SDA plates. Further identification of *Candida albicans* was done by germ-tube test and corn-meal agar.

**Result:** Overall prevalence of *Candida albicans* carriage was significantly higher and mean value of *Candida albicans* CFU (colony forming unit) was remarkably higher in group 1 (experimental group) as compare to group 2 (control group). Significant reduction in the frequency and mean value of *Candida albicans* CFU/plate was seen in children after treatment of carious lesions.

**Conclusion:** This study supports the active role of *Candida* species in dental caries. Hence, *Candida albicans* may play an important role as a risk factor for dental caries. It was also seen that the oral environment stabilization procedures were able to reduce *Candida albicans* counts. Thus, these procedures can be considered efficient in the reduction of caries risk.

**How to cite this article:** Srivastava B, Bhatia HP, Chaudhary V, Aggarwal A, Singh AK, Gupta N. Comparative Evaluation of Oral *Candida albicans* Carriage in Children with and without Dental Caries: A Microbiological *in vivo* Study. Int J Clin Pediatr Dent 2012;5(2):108-112.

## INTRODUCTION

Yeast is normally present in the oral cavity of healthy individuals and according to several studies, the percentage of *Candida* species colonization ranges from 20 to 40% in healthy individuals and becomes predominant flora in more than 60% of immunocompromised subjects.^1^ Besides iatrogenic factors, one factor that plays an important role in carriage is hostage. However, there is paucity of information on oral *Candida* in children.

A study by Russell and Lay (1973)^2^ demonstrated that the frequency of oral yeast carriage at birth was low, which doubled by the time the infants were discharged from the hospital about 7 days later and increased sharply after 1 month at home. Kleinegger (1996) indicated that the frequency of oral yeast carriage was 44% of the tested individuals in a group aged from 0.5 to 1.5 years, 24% in a 5 to 7 years group and 40% in a 15 to 18 years old group.^3^

Natural barriers against yeast colonization exist in body fluids and these barriers may vary as a function of age. Although these changes may be due to physiological factors related to age, changes in environment, and diet may also represent some of the important factors.^4^ Though caries prevalence is associated primarily with high levels of *Streptococcus mutans* and lactobacilli recovered from dentin and saliva, in recent years there has been an increased interest in the finding the relationship between oral fungal flora and dental caries.^5^

Dental caries is recognized as a bacterial disease affecting the mineralized tissues of teeth. However, *Candida* species too has been found to be associated with dental caries.^6^ The presence of *Candida* has been shown to enhance the adherence of *Streptococcus mutans* to the oral biofilm and carious tooth substance *in vitro*.^7^ These findings provide indirect evidence of association of *Candida* species with dental caries.

Hence, there was a need to evaluate the carriage of *Candida albicans* in children with dental caries and also to examine the presence of *Candida albicans* in extensive carious lesions before and after the treatment of the carious lesions.

## MATERIALS AND METHODS

This study was conducted on 60 childrens from 3 to 18 years of age who were selected from those attending the OPD of the Department of Pedodontics and Preventive Dentistry at Santosh Dental College and Hospitals, Ghaziabad. Out of these 30 patients were taken as experimental group I (with caries) and 30 patients as control group II (caries free). Each group was further divided into 3 subgroups, group A (deciduous), group B (mixed) and group C (permanent) according to their dentitions. Parents of selected patient were aware of the experimental design and had given their written informed consent before the study.

Inclusion criteria: Children with nursing bottle caries, Rampant caries, with more than 4 decayed teeth and sound periodontal conditions. Children who gave history of prolonged antibiotic treatment, fungal infection in the mouth, chronic diseases, such as diabetes and allergies, any developmental abnormality and less than 4 decayed teeth were excluded from the study.

For mycological studies, swab samples were taken from experimental group I and control group II. Swab sample was taken by rolling the sterile cotton swab across the dorsum of the tongue, vestibular sulcus and peak of the palatal vault. Swab sample was immediately smeared onto the surface of culture plate with Sabouraud's dextrose agar (with chloramphenicol 10%). Plates were aerobically incubated at 37° centigrade for 48 to 72 hours. Culture plates which were negative for Candidal growth were left on the bench for further 72 hours and then examined again before being discarded as negative.

Number of *Candida* colonies was determined by counting colony forming units on SDA plates. Preliminary identification of *Candida* was carried out after the growth of characteristic creamy convex yeast colonies on Sabouraud's dextrose agar. Smear was made for gram staining and was examined under microscope to recognize Gram-positive budding cells. Further species identification was done by germ tube test and cornmeal agar. Germ tube formation and chlamydospores formation was seen under microscope respectively in the case of *Candida albicans.*

### Statistical Analysis

The software used for the statistical analysis was SPSS (statistical package for social sciences) version 16.0. The values were represented in number (n), percentage (%) and mean (v). The statistical tests used were paired t-test for difference between the mean values and ANNOVA test for difference among the mean values of various subgroups. Chi-square test was used for comparison between the group. Comparison within in the various subgroups was done using post-hoc test (Bonferroni multiple comparisons test) and the level of significance was taken at 5% (p < 0.05).

## RESULT

It was observed that overall prevalence of *Candida albicans* in 60 subjects was higher (63.3%) as shown in Table 1. In Table 2, statistically significant difference was seen between the two groups (p-value < 0.05). The prevalence of *Candida albicans* carriage in subjects, with caries, i.e. in experimental group (group I) was significantly higher (93.3%) than in subjects without caries, i.e. control group (group II) (33.3%).

In Table 3, the mean value of *Candida albicans* colony forming unit (CFU) in SDA (Sabouraud's dextrose agar) plates in groups I and II was 25.37 ± 17.485 and 2.17 ± 3.312 respectively. Statistically significant difference was found between groups I and II (p-value < 0.05).

Frequency of *Candida albicans* was high in subjects with caries before treatment (93.3%) than the subjects after treatment (86.7%). Thus, significant reduction was seen between samples 1 and 2. Results are depicted in Tables 4 and 5 respectively. The mean value of *Candida albicans* colony forming unit in SDA plates in group I reduced significantly after treatment of carious lesions (Table 6) and its was also observed that the mean value of *Candida* colony count was high in primary and mixed dentition as compare to permanent dentition.

## DISCISION

Results of the present study showed high prevalence of *Candida albicans* carriage in children. Out of 60 childrens, 38 childrens were found positive for *Candida albicans* carriage (63.3%) which is in accordance with some previous studies Moalic et al (2001),^8^ MV Martini and GR Wilkinson (1983).^9^

**Table 1:** **Table 1:** Overall frequency of *Candida albicans* in 60 subjects (groups I and II)

*Subjects positive or negative for Candida albicans*		*Frequency (n)*		*Percentage*	
+ve		38		63.3	
–ve		22		33.7	
Total		60		100.0	

n: Number of subjects

**Table 2:** **Table 2:** Comparison between groups I and II for candidal growth using Chi-square test

*Groups*		*Number of subjects and* *percentage of candidal* *growth +ve/-ve*				*Total*		*Sig. (p-value)*	
		*+ve*		*–ve*					
Caries group I		28		2		30		0.000*	
		93.3%		6.7%		49.2%			
Caries free group II		10		20		30			
		33.3%		66.7%		50.8%			
Total		38		22		60			
		100.0%		100.0%		100.0%			

*(p-value < 0.05): There was a statistically significant difference b/w the two groups

**Table 3:** **Table 3:** Mean, standard deviation and test of significance of mean value of colony counts of *Candida albicans* in groups I and II (experimental and control groups )

*Swab sample*		*Groups*		*Mean*		*SD*		*Sig. (p-value)*	
Pretreatment		Caries group I		25.37		17.458		0.000*	
Swab sample in SDA plates		Caries free group II		2.17		3.312			

*(p-value < 0.05): Statistically significant

Statistically significant difference in the prevalence of *Candida albicans* carriage between the two groups (groups I and II) indicated that the state of oral hygiene and presence of dentinal carious lesion might be an important factor for the presence or absence of *Candida albicans* in the mouth of children as reported similarly by other authors (Moalic et al 2001, Rozkiewicz et al 2006).^6,8^ (Caterina signeritto 1992, Hodson JJ 1992, Coulter WA 1993).^1,10,11^

Mean value of colony forming unit in SDA (Sabouraud dextrose agar) plates was significantly high in group I (experimental group) as compared to group II (control group). Some authors have shown that *Candida albicans* might play a role as a risk factor for dental caries. For example, Moalic et al (2001)^8^ found that the carious teeth were more often present in subjects with abundance of *Candida albicans* and that seemed desirable since identification of *Candida albicans* is easier than that of *Streptococcus mutans*.

**Table 4:** **Table 4:** Frequency of *Candida albicans* in pretreatment sample 1 of experimental group (group I)

*Subjects positive or negative for Candida albicans in sample 1*		*Frequency (n)*		*Percentage*	
+ve		28		93.3	
–ve		2		6.7	
Total		30		100.0	

n: Number of subject

**Table 5:** **Table 5:** Frequency of *Candida albicans* in posttreatment sample 2 of experimental group (group 1)

*Subjects positive or negative for Candida albicans in sample 3*		*Frequency (n)*		*Percentage*	
+ve		26		86.7	
–ve		4		13.3	
Total		30		100.0	

n: Number of subjects

**Table 6:** **Table 6:** Mean standard deviation and test of significance of mean value of colony counts in swab samples 1 and 2 (experimental group)

*Swab sample in SDA plates*		*Mean*		*Std. deviation*		*Sig. (p-value)*	
Pretreatment sample 1		25.37		17.46		0.000*	
Posttreatment sample 2		12.60		9.34			

*(p-value < 0.05): There was a statistically significant difference b/w the two samples

M Raja et al (2010)^12^ in their study highlighted the positive correlation of *Candida albicans* with caries in mixed dentition. Same was observed in the present study. In the study by Russel and Lay (1973),^2^ the highest frequency of carriage of yeast was seen in primary dentition group (70%), followed by Mixed dentition group (56.4%) and in permanent dentition group 49.5%) was observed. With increasing age, the frequency of *Candida albicans* decreases. Same observation was seen in the present study. However, frequency of yeast carriage did not decrease because the carriage of non *Candida albicans* was increased.

The prevalence of *Candida albicans* in primary and mixed dentition group was significantly higher as compare to permanent dentition group in the present study. Strong association of *Candida albicans* and early childhood caries was seen. Prevalence of *Candida albicans* carriage was significantly high in subgroup A as compare to subgroup C. Most of the children with primary dentition had early childhood caries in the present study.

Hodson and Craig (1972)^10^ found that *Candida albicans* in the biofilm of ECC (56%) were twice more prevalent than that in caries free children (33%). Same observation was made in the present study, prevalence of *Candida albicans* carriage in group I (children with caries) was 90% and in group II (children without caries), it was found to be 40%. Likewise, Merchant et al 2001^14^ found that frequency of *Candida albicans* isolation were 89% in carious dentin of ECC (Early childhood caries) and 7% in the biolfilm of children without carious lesions.

Hossain et al (2003)^13^ observed that infants without caries harboured 2% of *Candida albicans* in saliva sample. In contrast, ECC group harbored the highest concentration of *Candida albicans*, 60% in saliva and 82% in carious lesion. Carvalho et al (2006)^14^ verified that frequency of *Candida albicans* was higher when compared in caries (14.3%) and caries free (12.5%) group.

The children with ECC have a frequent habit of night bottle feeding. Milk contains lactose, which is degraded to galactose and glucose, following addition of sucrose in baby bottle. Based on these findings Carvalho et al (2006)^14^ suggested that in ECC infant there is higher adhesion of *Candida albicans* due to presence of these sugars. Same observation was done in the present study as parents of most of the children in subgroup A gave history of bottle feeding upto 2 to 3 years of age. Thus, *Candida albicans* colonization in oral cavity of children might be related to pacifier usage, feeding habits and caries lesions.

According to some studies remotion of carious dentin and restoration of carious teeth seemed to reduce the colony counts of *Streptococcus mutans* and lactobacilli. But the study of the presence of *Candida albicans* after oral environment stabilization procedures (restorative and endodontic procedures) has been rarely discussed. The reduction in the number of colony counts of *Candida albicans* after oral environment stabilization procedures is expected as a result of the control of some predisposing factors that facilitates the colonization of this microorganism.^15^

In the present study, children affected by caries were treated and at the end of oral stabilization procedure, mean value of *Candida albicans* CFU (colony forming unit) in SDA (Sabourauds dextrose agar) plates significantly reduced. The mean value of *Candida albicans* CFU before treatment in group I was 25.37 ± 17.46 and after treatment of carious lesions mean value of *Candida* CFU in SDA plate was significantly reduced to 12.63 ± 9.54. Although, the carriage of *Candida albicans* was not completely eliminated after treatment. Four childrens out of 30 showed no candidal carriage in SDA plate.

The decrease in *Candida albicans* colonization after dental treatment was investigated by Mondin et al (2003) in 64 infants aged from 2 to 3 years, in whom 32 were caries free and 32 had caries. The operative intervention decreased the number of yeasts in ECC children. However, in the present study significant reduction of *Candida* colony counts was seen in primary, mixed and permanent dentition.

It was observed that the percentage and mean value of *Candida albicans* CFU (colony forming unit) was remarkably higher in group I (experimental group) as compare to group II (control group) highlighted the significant correlation between *Candida albicans* carriage and dental caries. Children who had ECC showed high prevalence of *Candida albicans* carriage as compare to children without ECC. Significant reduction in the frequency and mean value of *Candida albicans* CFU/plate was seen in children after the treatment of carious lesions.

Result of the present study supports the active role of *Candida* species in dental caries. Hence, *Candida albicans* may play an important role as a risk factor for dental caries.

The oral cavities of children therefore become one of the main sources of transmission of this fungus and its eradication should start with a reduction in its concentration in the mouth. The phenomenon of dental decay is a well known process and this is widely documented in the literature. This aspect however, is quite different in pediatric patients. Their mouths are often found to be in a very bad state, with widely destructive dental decay. So more emphasis should be given to prevention as it plays a crucial role in order to maintain a good health of the oral cavity of children.

Oral environment stabilization procedures have been used to reduce the number of pathogenic microorganisms in the mouth, preventing the installation of or progression of diseases. This procedure can create conditions for the improvement of oral health. Results show that oral environment stabilization is efficient in reducing *Candida* counts. The reduction could be probably due to the remotion of *Candida* colonization sites as proposed by Jacob et al 1998.

Dental caries is a major health problem with high prelevance, globally involving all regions and societies. Further work needs to be done on larger samples recruited in multicenteric or community based settings to substantiate the association of oral *Candida* carriage with dental caries. Future research also needs to focus on unraveling the precise mechanisms of Candidal involvement in the process of dental caries. This would provide us with insights into the complex pathogenesis of dental caries and open new avenues for its prevention and management.
